# Identification of Anti-Inflammatory Compounds from Hawaiian Noni (*Morinda citrifolia* L.) Fruit Juice

**DOI:** 10.3390/molecules25214968

**Published:** 2020-10-27

**Authors:** Dahae Lee, Jae Sik Yu, Peng Huang, Mallique Qader, Arulmani Manavalan, Xiaohua Wu, Jin-Chul Kim, Changhyun Pang, Shugeng Cao, Ki Sung Kang, Ki Hyun Kim

**Affiliations:** 1 College of Korean Medicine, Gachon University, Seongnam 13120, Korea; pjsldh@naver.com; 2 School of Pharmacy, Sungkyunkwan University, Suwon 16419, Korea; jsyu@bu.edu; 3 Daniel K. Inouye College of Pharmacy, University of Hawaii at Hilo, Hilo, HI 96720, USA; great7701@126.com (P.H.); mqader@hawaii.edu (M.Q.); arulmanim@gmail.com (A.M.); xiaohua3@hawaii.edu (X.W.); 4 Natural Product Informatics Research Center, KIST Gangneung Institute of Natural Products, Gangneung 25451, Korea; jckim@kist.re.kr; 5 School of Chemical Engineering, Sungkyunkwan University, Suwon 16419, Korea; chpang@skku.edu

**Keywords:** noni, *Morinda citrifolia*, anti-inflammation, NO production, RAW 264.7 cells

## Abstract

Noni (*Morinda citrifolia* L.) fruit juice has been used in Polynesia as a traditional folk medicine and is very popular worldwide as a functional food supplement. In this study, compounds present in Hawaiian Noni fruit juice, with anti-inflammatory activity in lipopolysaccharide (LPS)-stimulated RAW 264.7 cells were identified. Five compounds were isolated using a bioassay-driven technique and phytochemical analysis of noni fruit juice: asperulosidic acid (**1**), rutin (**2**), nonioside A (**3**), (2*E*,4*E*,7*Z*)-deca-2,4,7-trienoate-2-*O*-β-d-glucopyranosyl-β-d-glucopyranoside (**4**), and tricetin (**5**). The structures of these five compounds were determined via NMR spectroscopy and LC/MS. In an anti-inflammatory assay, compounds **1**–**5** inhibited the production of nitric oxide (NO), which is a proinflammatory mediator, in LPS-stimulated macrophages. Moreover, the mechanisms underlying the anti-inflammatory effects of compounds **1**–**5** were investigated. Parallel to the inhibition of NO production, treatment with compounds **1**–**5** downregulated the expression of IKKα/β, I-κBα, and NF-κB p65 in LPS-stimulated macrophages. Furthermore, treatment with compounds **1**–**5** downregulated the expression of nitric oxide synthase and cyclooxygenase-2. Thus, these data demonstrated that compounds **1**–**5** present in noni fruit juice, exhibited potential anti-inflammatory activity; these active compounds may contribute preventively and therapeutically against inflammatory diseases.

## 1. Introduction

Noni is the Hawaiian name given to *Morinda citrifolia* Linn. (Family: Rubiaceae), also known as beach mulberry or cheese fruit [1]. It is a small tropical evergreen tree or shrub that is native to Southeast Asia and is distributed in Australia and the South Pacific, including Hawaii [1,2]. The phytotherapeutic applications of noni fruit juice have been mentioned in folklore, and the juice has been successfully used as per traditional systems of medicine to treat a broad range of leading diseases like diabetes, cancer, hypertension, cardiovascular diseases, arthritis, pathogenic and worm infections, poor indigestion, and HIV–AIDS and drug addiction [3,4,5].

The products derived from noni are very popular globally, particularly the fruit juice, which is used as a functional food supplement [3]. Noni juice was introduced into markets in the USA in the 1990s and into European communities since 2003 [3]. By now, the products are very famous in New Zealand, Australia, and some Asian countries [6]. The worldwide market for noni-derived products is peaking each year; in 2006, it reached USD two billion [6].

**Figure 1:**
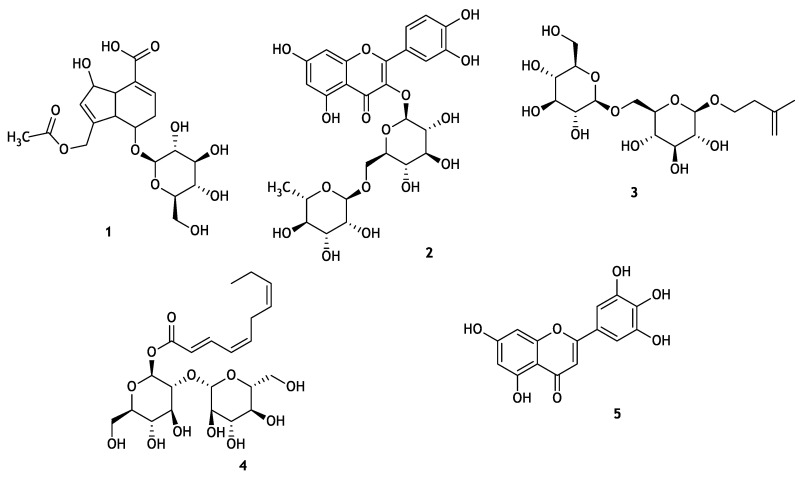
Chemical structures of compounds **1**–**5**.

Nuclear factor kappa B (NF-κB) is a transcription factor that mainly regulates the cellular responses to inflammation, cancer, and nervous system functioning [7]. The activation of NF-κB contributes to the development of various disorders, including rheumatoid arthritis, atherosclerosis, inflammatory bowel diseases, multiple sclerosis, and tumors [8]. The inhibition of NF-κB could stop the proliferation of tumor cells, which could become more sensitive to antitumor agents [9]. Thus, the inhibition of NF-κB signaling has potential therapeutic applications in cancer and inflammatory diseases [8]. It was reported that patients with stage III/IV invasive adenocarcinoma who consumed homemade noni fruit juice regained their body weight after 4 weeks [10]. This encouraged researchers to conduct in-depth studies on noni fruit juice. As an initiative in 2001, clinical studies have been carried out. However, after 5 years, they were unable to publish any data supporting the relevance [11]. In our previous studies on noni juice, we identified alkyl catechols (4-methyl catechol, 4-ethyl catechol, and 4-vinyl catechol) [12] and scopoletin [13], among which 4-methyl catechol, 4-ethyl catechol, and 4-vinyl catechol are potent molecules that provide protection against diseases, like cancer associated with oxidative stress, by stimulating nuclear factor erythroid 2-related factor 2 (Nrf2) transcription factors. In continuation, the present study was undertaken to isolate and identify inflammatory inhibitors present in noni fruit juice using a bioassay-guided technique. Five compounds (**1**–**5**) (Figure 1) were isolated using column chromatography and HPLC, and their structures were determined via NMR spectroscopic and LC/MS analyses. Here we report the isolation and structure characterization of these isolates found in noni fruit juice, as well as their anti-inflammatory effects and basic underlying mechanism of action.

## 2. Results and Discussion

### 2.1. Isolation and Identification of Bioactive Compounds from Noni Fruit Juice

**Figure 2:**
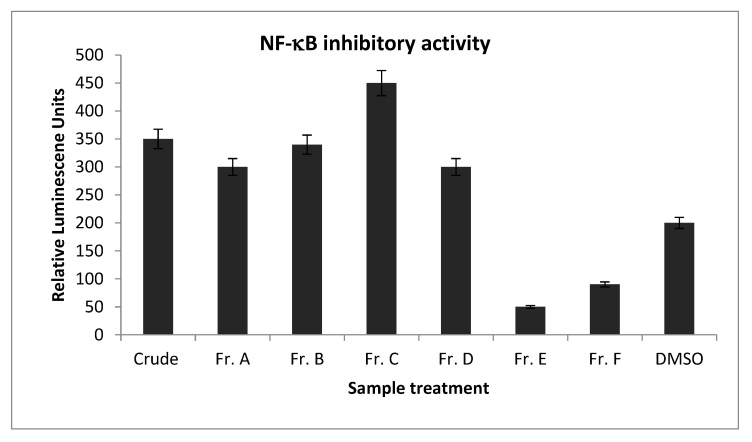
The effect of the crude extract (5 mg/mL) and the HP20 open column fractions (Fr. A–F, 5 mg/mL) of noni fruit juice on NF-κB in HepG2 cells. The negative control was 0.2% DMSO (mean ± SD).

**Figure 3:**
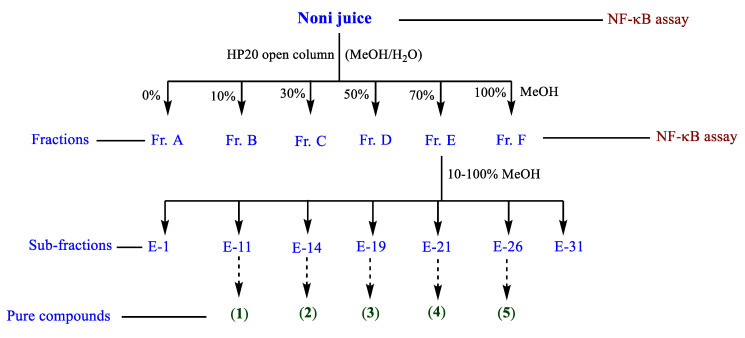
NF-κB assay-guided separation of compounds **1**–**5**.

Our preliminary study [13] showed that different noni juices, including purchased crude noni juice, ripe noni juice, and noni juice fermented with *Lactobacillus plantarum* (ATCC8014) at 22 °C (room temperature) at 24 h [13] demonstrated NF-κB inhibition. We decided to use the purchased noni juice in the anti-inflammatory assay because of its commercial availability, reproducibility and previously reported Nrf2 nuclear translocation activity [13]. Bio-assay guided fractionation method was used in this study [13,14,15,16]. The separation of the purchased noni juice via HP20 open column chromatography yielded six fractions (Fr. A–F), and Fr. E was active against NF-κB (Figure 2). Preparative HPLC separation followed by semi-preparative HPLC yielded five compounds: asperulosidic acid (**1**, *t*_R_ 18.0, 1 mg) [17], rutin (**2**, *t*_R_ 23.0, 3 mg) [18], nonioside A (**3**, *t*_R_ 21.2, 1 mg) [19], (2*E*,4*E*,7*Z*)-deca-2,4,7-trienoate-2-*O*-β-d-glucopyranosyl-β-d-glucopyranoside (**4**, *t*_R_ 21.0, 1 mg) [20], and tricetin (**5**, *t*_R_ 20.7, 3 mg) [21] (Figure 1 and Figure 3, see Supplementary Materials). The NMR data of the isolated compounds were consistent with previous reports.

### 2.2. NF-κB Inhibitory Activity of Compounds ***1**–**5***

The five isolated compounds (**1**–**5**) together with their parent fraction (Fr. E) were evaluated in a mammalian cell-based assay designed to monitor TNF-α-induced NF-κB activity. Compounds **1**–**5** showed NF-κB inhibitory activity with IC_50_ values of 12.8, 59.0, >100, 15.8 and 18.6 μg/mL, respectively, while the parent fraction (Fr. E) had an IC_50_ value of 205.7 μg/mL. When tested using the same conditions as the NF-κB assay, the five compounds (**1**–**5**, at 100 μg/mL each) and the parent fraction (Fr. E, at 1 mg/mL) did not exhibit any toxicity. The results demonstrated that compounds **1**–**5** possess anti-inflammatory properties. In the absence of cytotoxicity, inhibition of TNF-α-induced NF-κB activity indicates a chemopreventative potential.

### 2.3. Effects of Compounds ***1**–**5*** on Nitric Oxide Production

**Figure 4:**
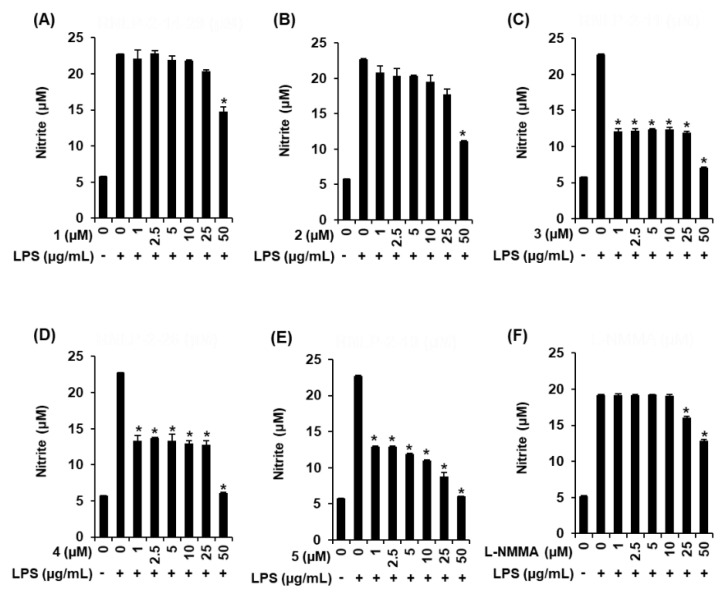
Comparison of inhibitory effect of the isolated compounds **1**–**5** and L-NMMA on LPS-induced nitric oxide (NO) production in RAW 264.7 macrophages. (**A**–**F**) The inhibitory effect of compounds on NO production was investigated using the Griess reagent (mean ± SD, * *p* < 0.05 compared to the LPS-treated group). In the graphs description, “+/-” means with/without LPS in the RAW 264.7 macrophages treatment for production of NO.

In the immune response, macrophages, as effector cells, detect pathogenic substances and are responsible for the inflammatory response [22]. Macrophages activated by lipopolysaccharide (LPS), one of the most potent activators of macrophages, release free radicals and inflammatory mediators [23]. The overproduction of inflammatory mediators such as nitric oxide (NO) has been correlated with inflammatory disease [24]. Many natural compounds have been identified as inhibitors of NO synthase and they have been reported to have potential anti-inflammatory effects. Polyphenolic Citrus sinensis fraction has been reported to inhibit pro-inflammatory mediators such as inducible nitric oxide synthase (iNOS) and cyclooxygenase-2 (COX-2), as well as NO [25]. In addition, some flavonoids are known to act as inhibitors of NF-κB, that plays a vital role in inflammatory responses [26]. According to the previous study, noni fruit juice is well-known to exhibit anti-inflammatory effects [27]. Administration of noni fruit juice alleviates inflammation symptoms of lung inflammation in rats [28] and a carrageenan-induced paw edema in rats [29]. These previous studies suggested that it is worthwhile to evaluate the anti-inflammatory effects of compounds isolated from noni fruit juice. Thus, to evaluate the anti-inflammatory effects of compounds **1**–**5**, their effects on NO production in LPS-activated RAW 264.7 macrophages were investigated. As shown in Figure 4A–E, compared with the LPS-alone treatment group (22.75 ± 0.05 μM), after coincubation of 50 μM of the compounds with LPS, the nitrite concentration was found to decrease by 14.73 ± 0.66 μM; 11.15 ± 0.09 μM with an IC_50_ value of 38.21 ± 0.44 μM; 7.11 ± 0.06 μM with an IC_50_ value of 2.01 ± 0.14 μM; 6.07 ± 0.13 μM with an IC_50_ value of 2.14 ± 0.61 μM; and 6.03 ± 0.02 μM with an IC_50_ value of 2.15 ± 0.03 μM, respectively, in compounds **1**–**5**. As shown in Figure 4F, after coincubation of 50 μM of the NO synthase inhibitor l-*N*^G^-nitroarginine methyl ester (L-NAME) with LPS, the nitrite concentration was found to decrease by 12.90 ± 0.09 μM compared with the LPS-alone treatment group (19.13 ± 0.14 μM). Compounds **2**–**5** were more efficient than L-NMMA (used as a positive control) in inhibiting NO production in LPS-activated RAW 264.7 macrophages. In the previous studies, asperulosidic acid (**1**) isolated from Hedyotis diffusa has been proven to inhibit NO production [30] and rutin (**2**) was also found to inhibit NO production [31]. Compound **5** was found to be the most effective compound in inhibiting NO production in LPS-activated RAW 264.7 macrophages. Previously, the anti-inflammatory effect of tricetin (**5**) has been evaluated in a mouse model of acute pulmonary inflammation induced by the LPS [32]. To investigate the mechanism underlying the inhibitory effects of compounds **1**–**5** on NO production in LPS-activated RAW 264.7 macrophages, Western blot analysis was performed.

### 2.4. Effects of Compounds ***1**–**5*** on the LPS-Induced Expression of IKKα/β, I-κBα, and NF-κB p65 in RAW 264.7 Mouse Macrophages

**Figure 5:**
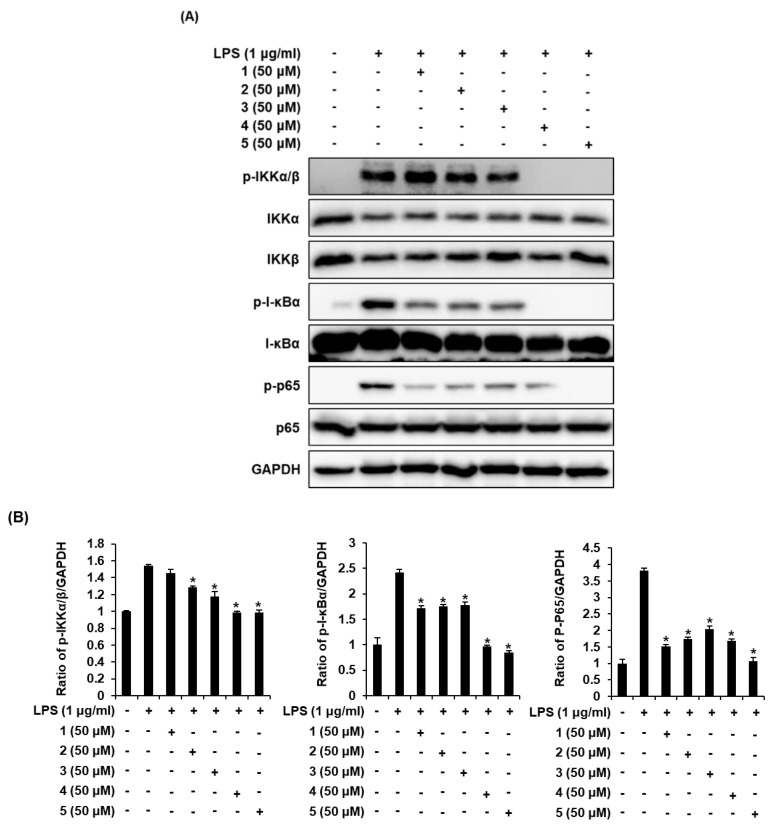
Comparison of effects of isolated compounds **1**–**5** on the LPS-induced protein expression of I-κBα, IκB kinase (IKK)α/β and NF-κB p65 in RAW 264.7 macrophages. (**A**) Representative Western blots for I-κBα, IKKα/β, NF-κB p65, and GAPDH protein expression. (**B**) Quantitative graph for Western blots (mean ± SD, * *p* < 0.05 compared with the LPS-treated group). In the graphs description, “+/-” means the protein expression of RAW 264.7 macrophages with/without LPS or sample (compounds **1**–**5**) respectively.

IκB kinase (IKK) contains two catalytic subunits including IKKα and IKKβ. The kinase is necessary for the phosphorylation of IκBα, which is associated with the activation of NF-κB [33]. NF-κB regulates the expression of inflammatory cytokine genes such as iNOS and COX-2. IKKα/β is activated by proinflammatory stimuli such as LPS [34]. Following the stimulation of RAW 264.7 cells with LPS, the effects of compounds **1**–**5** on the expression of IKKα/β, inhibitor of kappa B alpha (I-κBα), and NF-κB p65 were examined via Western blot analysis. As shown in Figure 5A, under unstimulated conditions, the cells did not express IKKα/β, I-κBα, and NF-κB p65; however, the expression of IKKα/β, I-κBα, and NF-κB p65 was increased considerably in response to LPS. Coincubation of the cells with compounds **4** and **5** dramatically inhibited the LPS-induced expression of IKKα/β, I-κBα, and NF-κB p65. Thus, compounds **4** and **5** inhibited the inflammatory response through NF-κB by interacting with IKKα/β and I-κBα in LPS-stimulated RAW 264.7 cells (Figure 5B).

### 2.5. Effects of Compounds ***1**–**5*** on the LPS-Induced Expression of iNOS and COX-2 in RAW 264.7 Mouse Macrophages

**Figure 6:**
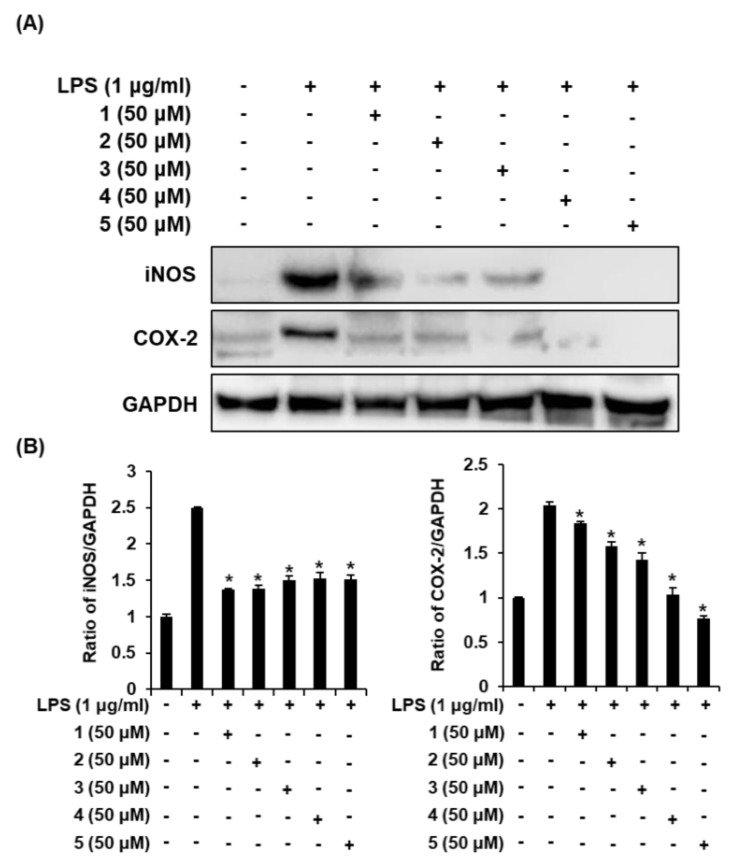
Comparison in effects of isolated compounds **1**–**5** (untreated (-) or treated (+)) on the LPS-induced protein expression of iNOS and COX-2 in RAW 264.7 macrophages. (**A**) Representative Western blots for protein expressions of iNOS, COX-2, and GAPDH. (**B**) Quantitative graph for Western blots (mean ± SD, * *p* < 0.05 compared with the LPS-treated group).

Inflammatory cytokine genes such as iNOS and COX-2 are coding genes regulated by NF-κB p65. These coding genes increase NO synthesis under the stimulation of inflammatory cytokines [35,36]. Quercetin isolated from noni fruit juice collected in Tahiti has been reported as an inhibitor of COX-2 [37]. In addition, in the rat chondrocytes, tricetin (**5**) was found to suppress production of NO and prostaglandin E_2_ (PGE_2_) and expression of iNOS and COX-2 [38]. Asperulosidic acid (**1**) and rutin (**2**) were reported to inhibit LPS-induced iNOS and COX-2 expression in RAW 264.7 macrophages [30,31], which displays an effect consistent with our study. However, the detailed molecular mechanisms of asperulosidic acid and rutin involved in the downregulation of iNOS and COX-2 in LPS-activated RAW 264.7 macrophages, have been previously unknown. In this study, the effects of compounds **1**–**5** on iNOS and COX-2 expression in RAW 264.7 macrophages were examined via Western blot analysis. As shown in Figure 6A, under unstimulated conditions, the cells did not express iNOS and COX-2; however, the expression of iNOS and COX-2 was greatly increased in response to LPS. Coincubation of the cells with compounds **1**–**5** dramatically inhibited the LPS-induced expression of iNOS and COX-2. Thus, compounds **1**–**5** regulated LPS-induced NO production through the inhibition of LPS-induced iNOS and COX-2 expression (Figure 6B). These results suggested that compounds **1**–**5** might be considered as a potential anti-inflammatory agent. However, the fact that the bioavailability and bioaccessibility of compounds **1**–**5** have not been verified is a limitation of this study, and the issue should be verified by additional studies including the results of animal experiments.

## 3. Materials and Methods

### 3.1. General Experimental Procedures

Thermo Scientific Ultimate 3000 preparative and semi-preparative HPLC systems equipped with a diode array detector (Germany) were used for the isolation of compounds using HPLC-grade MeOH (Fisher Chemicals, Hampton, NH, USA) and Milli-Q water as the mobile phases. 1D and 2D NMR data were recorded in deuterated methanol (Cambridge Isotope Laboratories Inc, Tewksbury, MA) on a Bruker 400 MHz NMR system, and LC-ESIMS data were obtained on an Agilent 1260 HPLC system coupled to a 6120 quadrupole LC-MS spectrometer in dual ionization modes (column: Phenomenex C18, 100 Å, 100 × 4.6 mm, 5 μm; flow rate: 0.2 mL/min; solvent: 10–100% acetonitrile–H_2_O with 0.1% formic acid, for 20 min).

### 3.2. Noni Juices

Commercially available noni juice was purchased from Virgin Noni Juice (http://www.virginnonijuice.com), order number 809979. Virgin Noni juice was prepared using a standardized traditional Hawaiian method known as “dripping.” First, the finest ripe noni fruits were carefully hand-picked, washed thoroughly with water, and placed in large containers. The juice was further fermented for several weeks to allow the juice to seep out of the fruits. Stacked noni fruits develop a natural positive pressure that further allows the juice to flow out under the weight of the ripe fruits [6]. The pure noni juice was further strained several times and pasteurized to preserve most nutrients, vitamins, and important active enzymes.

### 3.3. HP20 Open Column, Preparative, and Semi-Preparative HPLC Purification of Noni Fruit Juice Extract

Filtered noni juice (500 mL) was loaded into an open column packed with Diaion HP20 polymaric matrix (HP20 6.6 g, 1.5 × 6.0 cm) and eluted with MeOH–H_2_O (0%, 10%, 30%, 50%, 70%, and 100% MeOH) solvent systems. The resulting six fractions (A–F) were dried using SpeedVac. The fraction E (70%) was purified on a Thermo Scientific Ultimate 3000 preparative HPLC system (C18 column, 5 μm; 100.0 L × 21.2 mm i.d.; flow rate 10 mL/min; with 0.1% formic acid in mobile phases) and eluted with 10–100% MeOH–H_2_O to obtain 30 subfractions (E1–E30). Finally, in total, seven subfractions were obtained after combining them according to their chemical profiles (Figure 3). Further, a Thermo Scientific Ultimate 3000 semi-preparative HPLC system quipped with a HPLC/diode array detector [column: Phenomenex Luna C18 (100 Å, 250 × 10 mm L × i.d., 5 μm) at a flow rate of 3 mL/min] was employed to obtain pure compounds: asperulosidic acid (**1**, *t*_R_ 18.0, 1 mg/L), rutin (**2**, *t*_R_ 23.0, 3 mg/L), nonioside A (**3**, *t*_R_ 21.2, 1 mg/L), (2*E*,4*E*,7*Z*)-deca-2,4,7-trienoate-2-*O*-β-d-glucopyranosyl-β-d-glucopyranoside (**4**, *t*_R_ 21.0, 1 mg/L), and tricetin (**5**, *t*_R_ 20.7, 3 mg/L) from subfractions E-11, 14, 19, 21, and 26, respectively (Figure 1). In both HPLC systems, 210 and 254 nm wavelengths were used for the isolation. The structural characterization of the isolated compounds was performed using extensive analysis of 1D and 2D NMR and LC-MS data, along with published data.

### 3.4. NF-κB Assay

Human embryonic kidney cells HEK 293 (20 × 10^3^ cells/well) were maintained in Dulbecco’s modified Eagle’s medium (DMEM) (Invitrogen Co., New York, NY, USA) supplemented with 10% of fetal bovine serum (FBS), 100 units/mL of penicillin, 100 μg/mL of streptomycin, and 2 mM of l-glutamine and incubated for 48 h. After the incubation the culture media was replaced and treated with the test samples with different concentrations. Then the activator TNF-α (human, recombinant, *E. coli*, Calbiochem, San Diego, CA, USA) at 2 ng/mL was added and incubated for another 6 h, the spent media was removed and the cells were washed with phosphate-buffered saline. The cells were lysed on a shaker for 5 min using 50 mL of reporter lysis buffer (Promega, Madison, WI, USA) and were then stored at −80 °C. The luciferase assay was performed using the Luc assay system from Promega, USA and followed the manufacturer instructions. NF-κB inhibition was expressed in terms of IC_50_ values (i.e., the concentration required to inhibit TNF-α-induced NF-κB activity by 50%). The NF-κB inhibitor TPCA-1 was used as the positive control (IC_50_ 48.2 ± 0.8 nM) [39]. All experiments were performed in triplicate.

### 3.5. Cell Culture

RAW 264.7 mouse macrophages were purchased from the American Type Culture Collection (Rockville, MD, USA) and maintained in DMEM medium (Cellgro Thomas Scientific, Manassas, VA, USA) containing 1% penicillin/streptomycin, 10% FBS (Invitrogen Co., New York, NY, USA), and 4 mM l-glutamine in a 5% CO_2_ atmosphere at 37 °C.

### 3.6. Measurement of Cell Viability

RAW 264.7 cells were seeded in 96-well plates at 3 × 10^4^ cells/well. To determine the cell viability, cells were treated with compounds **1**–**5** for 24 h at 37 °C and further treated with Ez-Cytox solution for 40 min (Daeil Lab Service Co., Seoul, Korea) [40]. The absorbance values were measured at 450 nm using a PowerWave XS microplate reader (Bio-Tek Instruments, Winooski, VT, USA).

### 3.7. Measurement of NO Production

NO production was measured using Griess reagent (2% sulfanilamide, 5% phosphoric acid, and 0.2% naphthylethylenediamine dihydrochloride). *N*^G^-methyl-l-arginine acetate salt (L-NMMA; nitric oxide synthase inhibitor) was used as the positive control compound. RAW 264.7 cells were seeded on 96-well plates at 3 × 10^4^ cells/well. Then, the cells were treated with compounds **1**–**5** or L-NMMA for 24 h at 37 °C and further treated with LPS (1 μg/mL) for 24 h. The absorbance values were measured at 540 nm using a PowerWave XS microplate reader (Bio-Tek Instruments, Winooski, VT, USA).

### 3.8. Western Blot Analysis

Proteins in the samples (20 μg protein/lane) were separated by electrophoresis on a 10% sodium dodecyl sulfate–polyacrylamide gel and further transferred onto a polyvinylidene fluoride (PVDF) membrane. The PVDF membrane was incubated with primary antibodies (Cell Signaling, Beverly, MA, USA) against IKKα, phospho-IKKα/β, IKKβ, I-κBα, phospho-I-κBα, NF-κB p65, phospho-NF-κB p65, iNOS, COX-2, and GAPDH for 1 h at room temperature. After binding with HRP-conjugated anti-rabbit antibodies (Cell Signaling, Beverly, MA, USA) for 1 h, the PVDF membranes were developed using enhanced chemiluminescence (ECL) Advance Western Blotting Detection Reagents (GE Healthcare, Little Chalfont, UK) and visualized using a FUSION Solo Chemiluminescence System (PEQLAB Biotechnologie GmbH, Germany).

### 3.9. Statistical Analysis

Statistical significance was assessed using a one-way analysis of variance (ANOVA) and multiple comparisons with a Bonferroni correction. A *p* value < 0.05 indicated statistical significance. All analyses were conducted using SPSS Statistics ver. 19.0 (SPSS Inc., Chicago, IL, USA).

## 4. Conclusions

**Figure 7:**
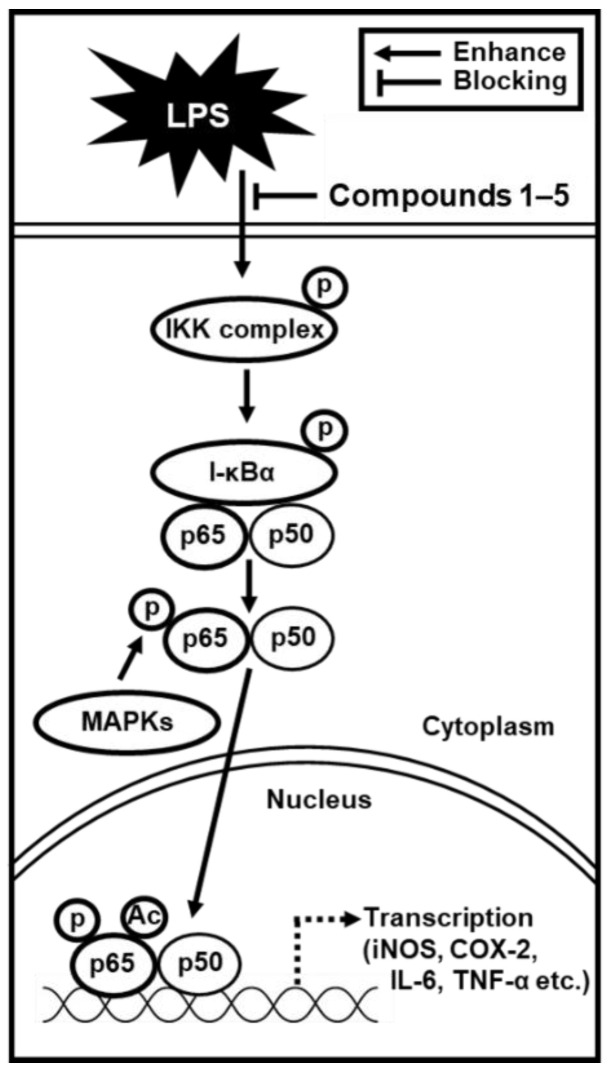
Schematic pathway of the role of isolated compounds **1**–**5** in inflammatory responses.

We demonstrated that compounds **1**–**5,** present in noni fruit juice, inhibited the LPS-induced inflammatory response in RAW 264.7 macrophages. The anti-inflammatory effects were determined to be mediated through the IKKα/β, I-κBα, and NF-κB p65 signaling pathways, which activate iNOS and COX-2 and regulate NO production (Figure 7). Our findings reveal the mechanisms underlying the anti-inflammatory action of compounds from noni fruit juice and their potential therapeutic use against inflammation-related disorders. Furthermore, this study provides supporting evidence that noni fruit juice can be a potential anti-inflammatory agent against inflammation-related disorders.

## Supplementary Materials

The Supplementary Materials are available online, Figure S1: ^1^HNMR spectrum of compound **1** (in MeOH-*d*_4_); Figure S2: ^1^H-NMR spectrum of compound **2** (in D_2_O-*d*_2_); Figure S3: HSQC spectrum of compound **2** (in D_2_O-*d*_2_); Figure S4: ^1^H-NMR spectrum of compound **3** (in MeOH-*d*_4_); Figure S5: ^1^H-NMR spectrum of compound **4** (in MeOH-*d*_4_); Figure S6: ^1^H-NMR spectrum of compound **5** (in MeOH-*d*_4_).
