# Supplementary material for: Identification of Anti-Inflammatory Compounds from Hawaiian Noni (Morinda citrifolia L.) Fruit Juice
Source: Molecules. 2020 Oct 27;25(21):4968. doi: 10.3390/molecules25214968 (PMC7662328; doi:10.3390/molecules25214968)
Supplement: Supplementary file 1 [file molecules-25-04968-s001.pdf]

Supplementary Materials: Identification of Anti-inflammatory Compounds from Hawaiian Noni (*Morinda citrifolia* L.) Fruit Juice

Dahae Lee[1,†], Jae Sik Yu[2,†], Peng Huang[3], Mallique Qader[3], Arulmani Manavalan[3], Xiaohua Wu[3], Jin-Chul Kim[4], Changhyun Pang[5], Shugeng Cao[3,*], Ki Sung Kang[1,*] and Ki Hyun Kim[2,*]

1 College of Korean Medicine, Gachon University, Seongnam 13120, Republic of Korea; pjsldh@naver.com (D.L.)
2 School of Pharmacy, Sungkyunkwan University, Suwon 16419, Republic of Korea; jsyu@bu.edu (J.S.Y.)
3 Daniel K. Inouye College of Pharmacy, University of Hawaii at Hilo, Hilo, Hawaii 96720, United States; great7701@126.com (P.H.); mqader@hawaii.edu (M.Q.); arulmanim@gmail.com (A.M.); xiaohua3@hawaii.edu (X.W.)
4 KIST Gangneung Institute of Natural products, Natural Product Informatics Research Center, Gangneung, 25451, Republic of Korea; jckim@kist.re.kr (J.C.K.)
5 School of Chemical Engineering, Sungkyunkwan University, Suwon 16419, Republic of Korea; chpang@skku.edu (C.P.)

† These authors contributed equally to this work

Figure S1. ^{1}H-NMR spectrum of compound **1** (in MeOH-d_4)

Figure S2. ^{1}H-NMR spectrum of compound **2** (in D_2O-d_2)

Figure S3. HSQC spectrum of compound **2** (in D_2O-d_2)

Figure S4. ^{1}H-NMR spectrum of compound **3** (in MeOH-d_4)

Figure S5. ^{1}H-NMR spectrum of compound **4** (in MeOH-d_4)

Figure S6. ^{1}H-NMR spectrum of compound **5** (in MeOH-d_4)

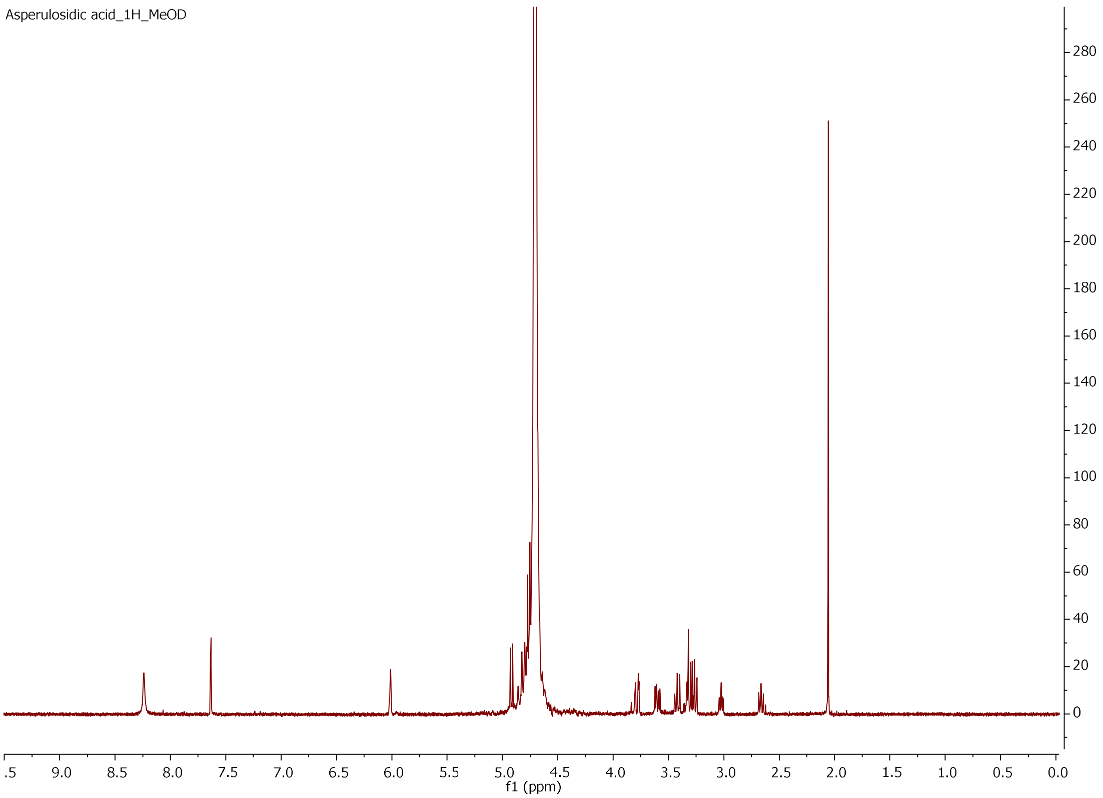

Figure S1. ^{1}H-NMR spectrum of compound **1** (in MeOH-d_4)

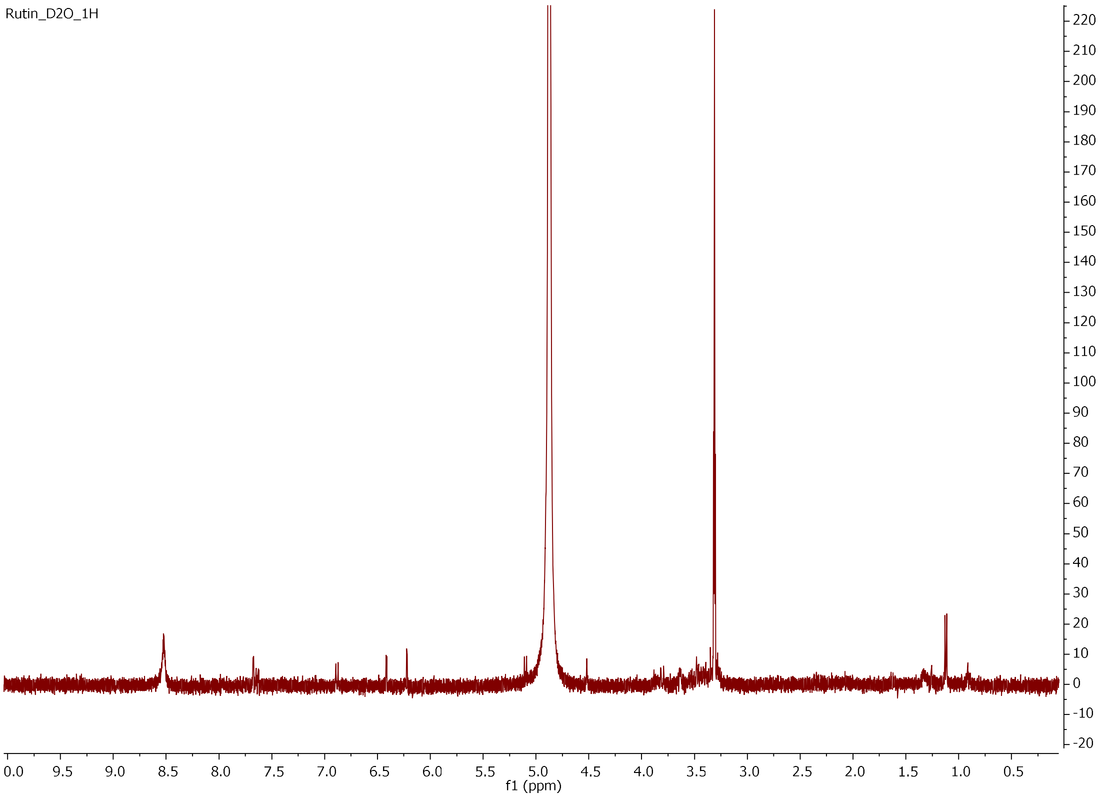

Figure S2. ^{1}H-NMR spectrum of compound **2** (in H_2O-d_2)

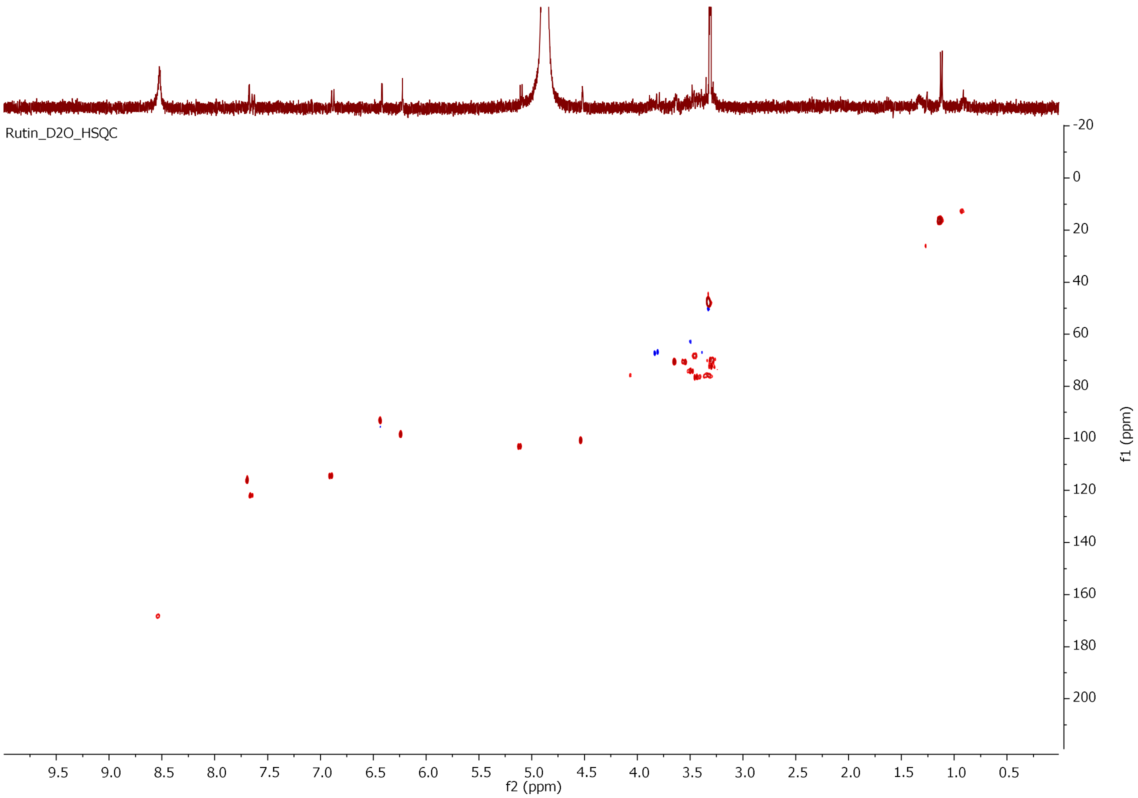

Figure S3. HSQC spectrum of compound **2** (in H_2O-d_2)

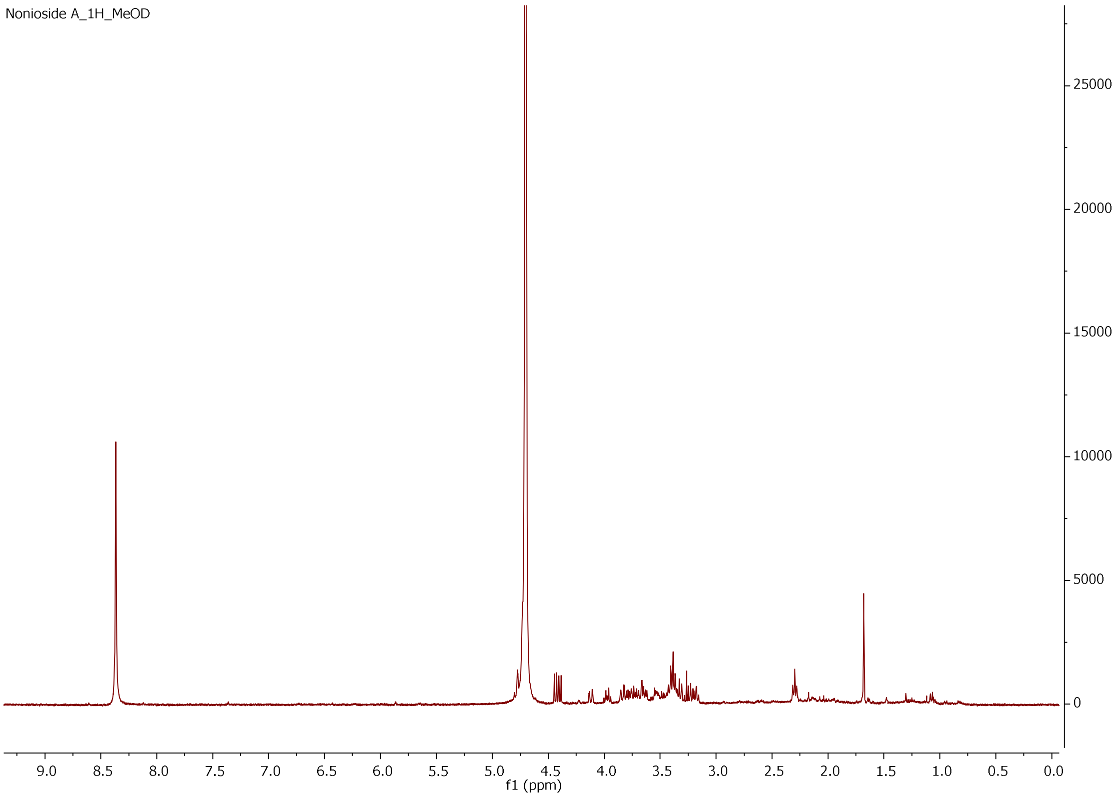

Figure S4. ^{1}H-NMR spectrum of compound **3** (in MeOH-d_4)

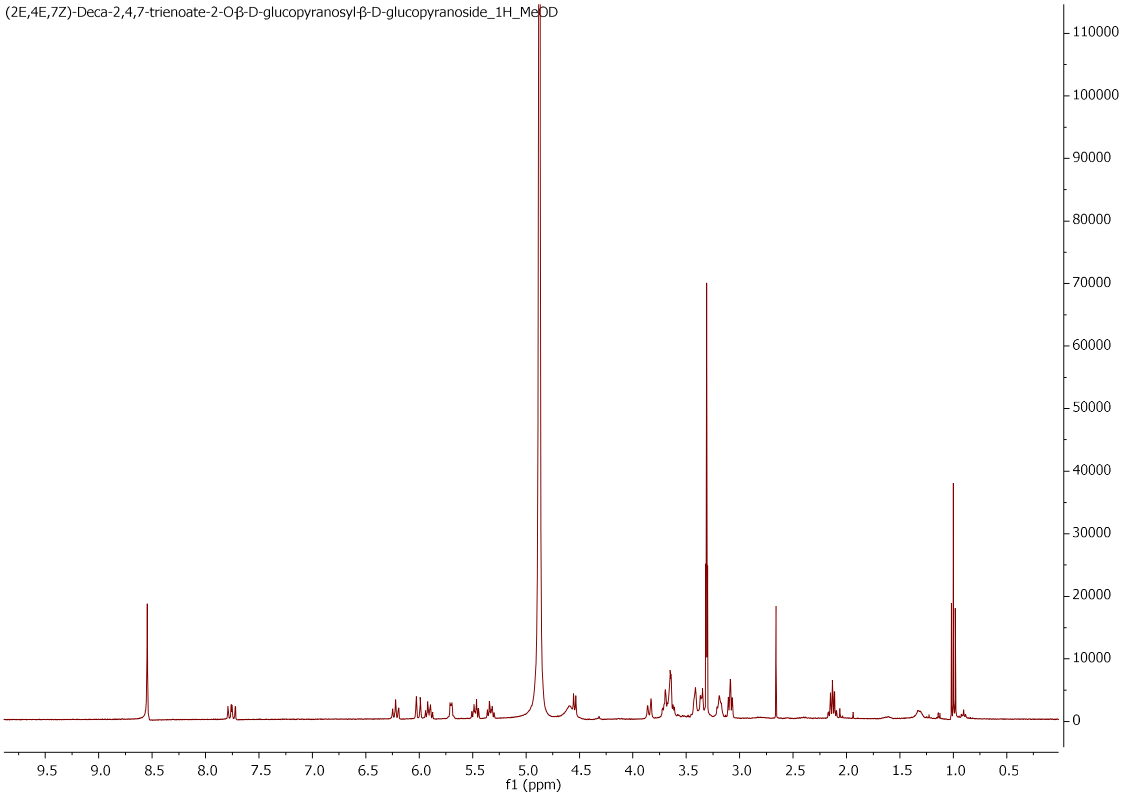

Figure S5. ¹H-NMR spectrum of compound **4** (in MeOH-d_4)

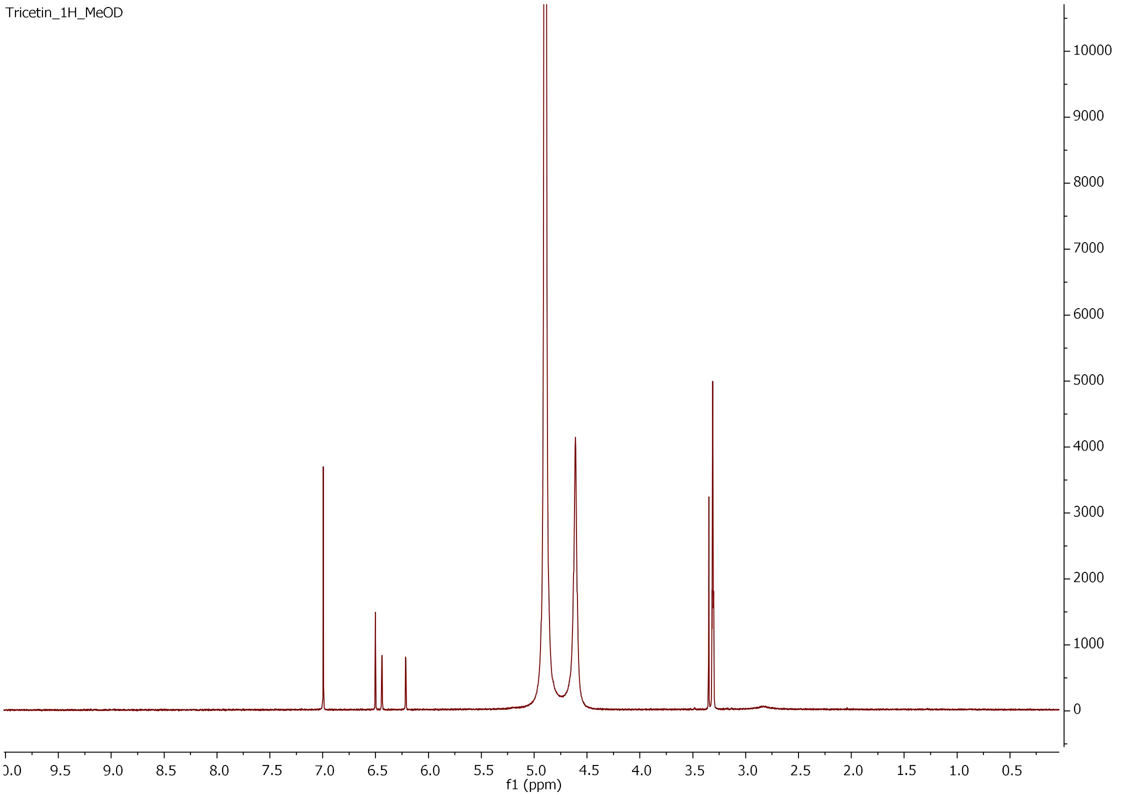

Figure S6. ¹H-NMR spectrum of compound **5** (in MeOH-d_4)
